# An Investigation of Knee Injury Profiles among Iranian Elite Karatekas: Observations from a Cross-Sectional Study

**DOI:** 10.3390/ijerph18136888

**Published:** 2021-06-27

**Authors:** Hamidreza Naserpour, Julien S. Baker, Amir Letafatkar, Giacomo Rossettini, Frédéric Dutheil

**Affiliations:** 1 Department of Biomechanics and Sport Injuries, Kharazmi University, Tehran 15875-4398, Iran; Hamidreza.Naserpour@gmail.com; 2 Centre for Health and Exercise Science Research, Hong Kong Baptist University, Hong Kong, China; jsbaker@hkbu.edu.hk; 3 School of Physiotherapy, University of Verona, Via Bengasi 4, 37134 Verona, Italy; giacomo.rossettini@gmail.com; 4 Physiological and Psychosocial Stress, UMR CNRS 6024 LaPSCo, University of Clermont Auvergne, 63000 Clermont-Ferrand, France; frederic.dutheil@unicaen.fr; 5 Preventive and Occupational Medicine, University Hospital of Clermont-Ferrand, 63000 Clermont-Ferrand, France; 6 Wittyfit, 75009 Paris, France

**Keywords:** knee, martial arts, injury mechanism, epidemiology

## Abstract

Karate training, despite the many positive health benefits, carries a risk of injury for participants. The current cross-sectional study aimed to investigate knee injury profiles among Iranian elite karatekas. Participants who attended the national team qualifiers, which included 390 male Kumite karatekas (age 24 ± 3 years old and weight 63 ± 12 kg), participated in this study. Information on knee injuries (injury history, type of injury mechanisms, and effects of knee symptoms on the ability to perform daily activities and recreational activities) were obtained using the Knee Outcome Survey (KOS). Using Pearson’s correlation coefficient, the study examined the relationships between different variables, including KOS subscales and levels of self-reported knee joint function. Our findings indicated that 287 karatekas (73.6%) experienced knee injuries. The anterior cruciate ligament (ACL) rupture (6.9%), articular cartilage (5.4%), and meniscus damage (3.8%) were the main typology of injury. In addition, there were no differences in knee injuries between the non-dominant and dominant legs. Most injuries occurred during the preparatory period (*n* = 162, 50%), especially during training periods. The KOS subscales scores (Mean ± Sd) for activities of daily living (ADL) and sports activity (SAS) were, respectively, 89 ± 11 and 91 ± 9. The self-reported scores for both the ADL and SAS subscales were, respectively, 89 ± 11 and 90 ± 10. Pearson coefficients of ADL and SAS subscales with their self-reported score were r = 0.761 (*p* < 0.0001) and r = 0.782 and (*p* < 0.0001), respectively. The profile of knee injuries in the current investigation is similar to previous surveys that reported lower extremity injury patterns. The findings of this study could be adopted to inform practice aimed at planning interventions for the reduction and prevention of knee injuries among karatekas.

## 1. Introduction

Martial arts originated from China, Korea, and Japan and include techniques for fighting using different parts of the body. The most important benefits of these exercises are increases in physical fitness, self-defense, flexibility, and self-confidence [1,2]. The martial arts have gained popularity over the years, and these eastern sports are now practiced all over the world [3]. Karate is recognized as one of the most popular martial arts in the world, and its popularity continues to increase in Iran [4,5,6]. Karate competitions comprise two elements called kata (form) and Kumite (fighting). Kumite is an Olympic-style karate competition involving active movements that require maximum intensity to perform [7,8,9]. Combat sport activities are not entirely safe, and there are risks of injury for those involved in participation, so the incidence of injury in karate is inevitable. The incidence of injuries during sports performance is a barrier to engaging in sports activities that limits individual levels of participation [10]. Athlete exposure to the potential dangers of sport can be diminished if they are aware of the biomechanical injury mechanisms, consequences of injuries, and treatments that prevent and minimize future injury [11].

According to previous epidemiological studies, the most common injury sites among karatekas are, respectively, the trunk and neck (40%), the knee joint (32%), upper extremities (16%), and the trunk in isolation (12%) [4,12]. Lower extremities are the most frequent sites of injuries in other martial arts, such as taekwondo (57%) and judo (39%) [13]. In martial arts, noticeable pressure is applied to the lower extremities of karatekas because of the repeated strikes, jumping movements, and landings from height [9].

The lack of awareness of the problem among karatekas can be considered the most crucial risk factor in the occurrence of karate injuries [14,15]. Thus, comprehensive and scientific planning is required to prevent injuries from occurring among karatekas [16]. The initial step for enhancing safety in karate is problem recognition. Subsequently, preventive measures can be introduced to reduce injury prevalence [11,14]. Injury prevention strategies can change and minimize the profile of injuries by using effective interventions [14]. Since the profile of injury in karate is both specific and variable, preventive measures should be introduced to prevent injury as required [17].

It is essential for coaches, athletes, and physicians that the body parts most affected in terms of injury are identified [18,19]. This would result in treatment and training strategies that could minimize injury effects. Therefore, this study aimed to investigate knee injury profiles in Iranian male elite karatekas.

## 2. Materials and Methods

### 2.1. Study Design and Ethical Issue

A cross-sectional survey was performed in January 2019, following the Helsinki Declaration regulations regarding research on human subjects [20], and ethical procedures were approved by the Research Ethics Committee of the Sports Science Institute with the code: IR.SSRI.REC.1400.1070. The method section was reported according to Strengthening the Reporting of Observational Studies in Epidemiology (STROBE) [21].

### 2.2. Participants and Setting

Members of the Iranian National Karate Federation, 390 male karatekas (Mean ± Sd: age 24 ± 3 years old, weight 63 ± 12 kg and height 172 ± 10 cm) Fighting Style (Kumite) who participated in the national competition volunteered as participants for this study. The national competition was held in Shiraz city-Iran.

### 2.3. Questionnaire Development

Our survey was composed of the following questionnaires adopted to collect data:

(A) Questionnaire on Demographic and Sports information: including demographic features, such as height, weight, age, exercise history, the number of training sessions per week, training days and hours, duration of the training, and warm-up methodologies. Questionnaire (A) is enclosed in the paper as Supplementary Materials (NO = 1).
(B) *Questionnaire on Knee Injury Profile*: the history of injury; injured leg (dominant or non-dominant); injury mechanisms; injury time, treatment, and type of aftercare. Injury incidence was determined as Σ injuries/Σ exposure hours × 1000. Questionnaire (B) is enclosed in the paper as Supplementary Materials (NO = 2).
(C) *Knee Outcome Survey (KOS*): The KOS is a self-reported questionnaire used to determine the amount of knee joint disability during daily activity and exercise [22,23]. It has been translated into the Persian language [24]. KOS is widely used for the evaluation of various knee injuries, including meniscus damage, ligament, cartilage lesion, patellofemoral pain syndrome, knee dislocation, or osteoarthritis [25,26] in both athletic and elderly populations [27,28].

The KOS assesses the effects of knee disorders by using five subscales: pain and symptoms in daily activity living scale (ADL); pain and symptoms using the sports activity scale (SAS), and knee-related quality of life scale (QOL). The KOS has a 42-item disease-specific questionnaire with a five-point Likert scale ranging from 0 to 4 (extreme problems/disability). Raw scores, also transformed into a 0–100 scale, with 0 showing extreme problems and 100 showing no problems, were calculated for each subscale separately [23]. These sub-scales have high validity and reliability for ADLS (0.97, 0.78) and SAS (0.97, 0.97), respectively [14,24,25,26].

After the compilation of the KOS questionnaire, karatekas were also asked to score their knee function during daily life and sport activity with a visual analog scale from 0 (extreme problem) to 100 (no problem). This score was assumed as a self-reported scale, which shows the presumption of motor consciousness [14]. Questionnaire (C) is enclosed in the paper as Supplementary Materials (NO = 3).

### 2.4. Data Collection Procedure

The information and purpose of the survey were explained by the researcher to all karatekas before completion and data collection, and all participants completed informed consent forms. This self-explanatory survey was administered using a paper and pencil approach [29] which takes approximately 10 min to complete [22]. The researcher was also available to answer participants’ questions and clarify any ambiguities.

### 2.5. Data Analyses

The recorded data was exported to SPSS spreadsheets, and questionnaire data were excluded in the survey if over 20 percent of the data were missing [30]. For descriptive statistics, all questions with only one option are presented using descriptive statistics (mean ± standard deviations—Mean ± SD), and other continuous variables are presented using Confidence Intervals at 95% level (95%CI). The Pearson correlation coefficient set at a significance level of 0.5 was used to identify any correlation between the KOS sub-scales during daily (ADL) and sport (SAS) activities, with a corresponding self-reported visual analog score using SPSS software version 25 for Windows (SPSS, Inc., Chicago, IL, USA).

## 3. Results

### 3.1. Participants’ Characteristics and Sport Backgrounds

Participants included 520 karatekas, of which 75% (*n* = 390) responded to the survey. Among them, 287 karatekas (73.6%) reported sustaining one or more knee injuries. The average demographical data of karatekas were respectively: age—24 years old (95%CI 23.7–24.3); weight—63 kg (95%CI 61.8–64.2); and height—172 cm (95%CI 171–173).

Table 1 provides the background detail of the participants. The findings revealed that approximately seventy percent of karatekas had over five years of karate experience and twenty-five percent competed in the national category.

Results also showed that karatekas have over 3 days and session exercise per week. Data showed that over 75% of participants have at least a 15–30 min warm-up protocol, including stretching, running, and combat exercises.

### 3.2. Participants’ Knee Injury Profiles

**Figure 1:**
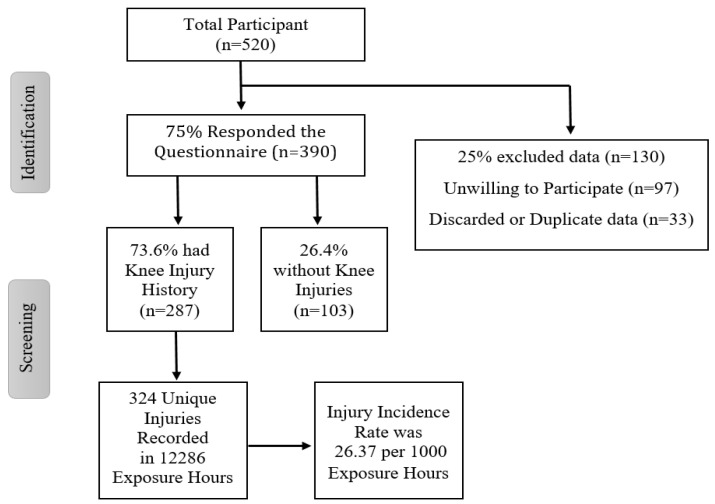
Flowchart of participants included in the study.

There were 324 injuries sustained in 12,286 exposure hours, resulting in an overall injury incidence of 26.37 per 1000 match training hours. Figure 1 illustrates a flowchart of participants in the study.

It should be noted that further analyses presented here, included the karatekas who had a history of injuries, and other healthy participants were excluded in data distribution. The outcomes of the Knee Injury profile questionnaire indicated that the frequently injured limb among athletes with history of injuries was the dominant leg (dominant—*n* = 103, 26.4%; non-dominant—*n* = 95, 24.4%; both *n* = 89, (22.8%), and mostly occurred during training (*n* = 195, 50%); competition (*n* = 55, 14.1%); and both (*n* = 37, 9.5%).

**Figure 2:**
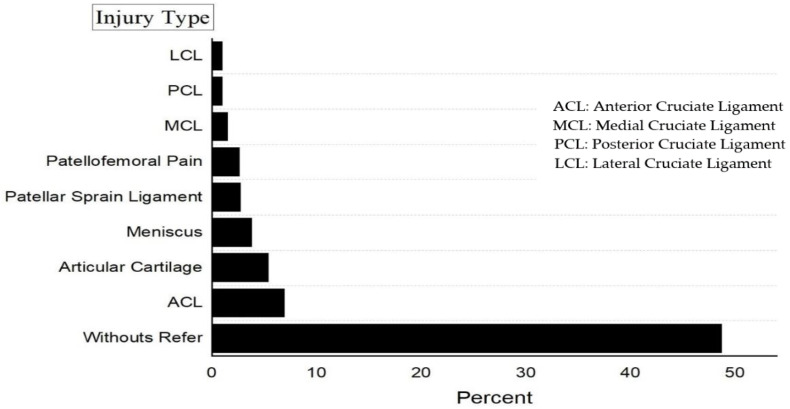
The knee injuries distributions among participants.

The most common treatment methods used were self-treatments (*n* = 130, 33.3%), physiotherapy (*n* = 56, 14.4%), and use of other self-medication techniques (*n* = 25, 6.4%). Seventy-one athletes (18.2%) did not use any treatment, and three participants (0.8%) used knee braces or had surgery. Further injury information is presented in Figure 2.

The mechanisms of injury reported by participants were hitting the opponent (*n* = 72, 41.1%), abrupt rotation of the feet (*n* = 63, 16.5%), landing from height (*n* = 35, 9.1%), abrupt stopping (*n* = 14, 3.6%), and falling (*n* = 13, 3.3%).

Karatekas resumed their training only one week after injury (*n* = 185, 47.4%). There were only 11.8% of contestants (*n* = 46) who had over 21 days of recovery time before returning to competition.

### 3.3. Participants’ KOS Subscales and Self-Reported Score

**Figure 3:**
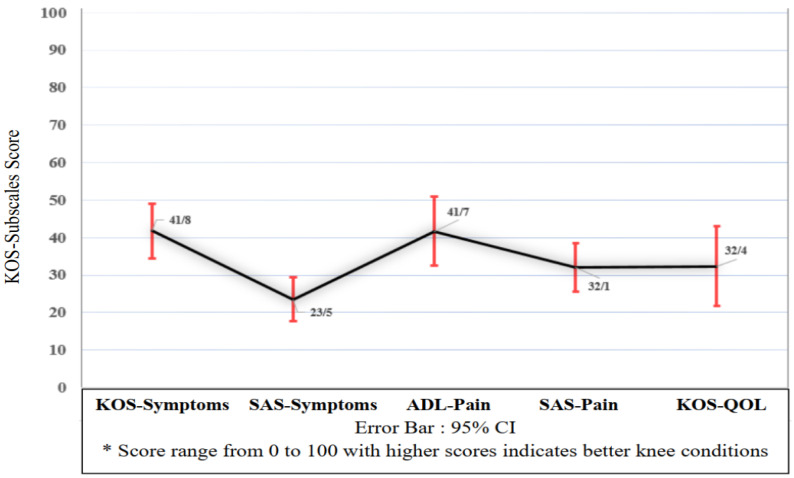
KOS-Subscales Score related to the quality of life, pain, and symptoms in daily and sports activities. Abbreviation: KOS: Knee Outcomes Survey, SAS: Sport Activity Scale, ADL: Activity of Daily Living, QOL: Quality of Life.

The KOS scores for ADL and SAS subscales were, respectively, 89 ± 11 and 91 ± 9. The self-reported scores for ADLs and SAS subscales were respectively 89 ± 11 and 90 ± 10. Pearson coefficients for ADLs and the SAS subscales with their self-reported score were r = 0.761 (*p* < 0.0001) and r = 0.782 (*p* < 0.0001), respectively. The knee-related quality of life of karatekas was 32.04 ± 10.6, which can be concluded to be in a moderate range. KOS-Subscales Score (95% CI) is presented in Figure 3.

## 4. Discussion

This study aimed to investigate the profile of knee injuries in Iranian elite karatekas. Our main finding was that 73.6% of participants (*n* = 287) experienced knee injuries. Our data were compatible with the studies of Piejko et al. (2019) and Brito Antonio et al. (2019), who demonstrated that the lower extremity and the knee were the most prevalent sites for injuries recorded in karatekas [31,32].

The most frequent mechanism of injury was hitting the opponent (*n* = 72, 41.1%). In 2015, the world karate federation implemented new rules. Accordingly, if karatekas apply a hand technique, the referees award the fighters with 1 point, but a leg technique has a value of 2 or 3 points. Thus, athletes are more eager to use their dominant lower body striking ability, which leads to more lower extremity injury [8,33,34].

A quarter of the karatekas competed at the national level, and 70% of them had more than 5 years of karate experience, which indicated that they were qualified enough to be nominated as professionals [33], representing an element of longevity compared to previous research [35].

Data also indicated that during the warm-up exercises, over 25% of participants refused to perform stretching exercises. It has been well documented that warm-up and flexibility exercises play an essential role in reducing injuries [14,36], highlighting the need for explaining to karatekas the importance of incorporating stretching into their training routine.

Most injuries occurred in the preparatory phase (50%) and mainly occurred in training sessions. Karatekas spent most of their time in the preparatory phase (67%, 1–2 h per session), and 75% of them participated in over three sessions per week. This finding agrees with the results of Brito Antonio et al. (2019) and Mahdavi et al. (2017) [14,32], suggesting that coaches need to pay specific attention to this phase of training.

The results revealed that only 12% of participants had acute injuries that led to missing over 21 days of training, and about half of them (47.8%) resumed their training only one week after injury. This finding supports Arriza et al. (2005) and Sharma et al. (2001), who reported fewer injuries as a result of actual participation in karate competition compared to other sports [16,37]. On the other hand, one-third of karatekas reported self-treatments, and 18% did not refer to a physician to alleviate the symptoms. Reducing the duration of the recovery period without specialized injury prevention and rehabilitation training could lead to re-injury and have a detrimental effect on the competitor’s health [15]. It is essential that rehabilitation and training be sufficiently vigorous to prepare the injured area for the demands of the competition. With each increase in activity, the pain may contribute to a reversal to an endurable level of sports performance and chronic injuries [38].

The results highlighted that ACL rupture (6.9%), articular cartilage (5.4%), and meniscus damage (3.8%) had the highest prevalence of injury. ACL, articular cartilage, and meniscus injuries are frequent injuries in sports activities that are associated with pivoting, decelerating, twisting, and jumping [39], which are frequently used movements in karate [9]. Our findings would be useful in informing coaches and clinicians about the necessity to utilize specialized preventive activities aimed at reducing the risk of ACL injury during training [40].

In addition, the results of the knee performance scale and self-reported scores emerging from karatekas’ knee function for ADL and SAS revealed high motor consciousness in the knee joint, which has not been mentioned in previous studies. Skinner et al. (1991) reported that motor consciousness weakness is a potent factor in the etiology of knee injuries (e.g., meniscus) and contributes to degenerative articular disease [41]. Thus, future studies should investigate the motor consciousness in the knee joint as an element of interest to help prevent the occurrence of injuries among karatekas.

Finally, limitations in daily functional activities (KOS-ADLs) were considerably higher than sport skills (KOS-SAS), and the most common complaint was sitting with a bent knee. It seems that this may be related to meniscus damage, knee joint stress, a range of motion limitation, or a combination of all three [42,43].

### Strength and Limitation

The current study provides novel findings and outlines knee injury profiles adding to the literature and existing research on karate fighters. The study also examined large numbers of elite karatekas. As far as we are aware, this is the first study with many professional participants providing knee injury profiles of karate Olympic style (Kumite). This study also had a larger sample of participants in comparison to other investigations, such as Rahnama et al. (*n* = 140) or Dadgar et al. (*n* = 45) [18,33].

As a limitation, we adopted a convenience sampling style. Moreover, there were limitations in data collection due to the voluntary contribution of participants. Thus, approximately 25% of the competition karatekas refused to co-operate with the research team, and this may have affected outcomes. A further limitation was the unisex of participants. This methodological choice was due to a limited number of female karatekas. Moreover, the Iranian officials of the federation that promoted the competition oppose the presence of male researchers among female participants because of common religious beliefs. This made data collection on the group virtually impossible, and further studies should include female researchers to alleviate this problem.

## 5. Conclusions

Over 70% of the karatekas had knee injuries. The anterior cruciate ligament was the most prevalent injury among the fighters. The findings obtained could help devise strategies to minimize injury by prescribing specialized training programs for karateka using an injury prevention approach. It is recommended to expand this research in different countries (e.g., Europe, America, Asia), including female populations, to identify how the differences in gender could contribute to the injury profiles obtained.

## Figures and Tables

**Table 1:** Participants’ sports background information.

**Sports Background Variable**	**Frequency**	**Sports Background Variable**	**Frequency**
Sport Experience	N (%)	Sport Level	N (%)
≤3 years	5 (1.3%)	National	98 (25.1%)
3–5 years	113 (29%)	Club	292 (74.9%)
≥5 years	272 (69.7%)	Collegiate	-
Sessions Per Week	**N (%)**	**Days per Week**	**N (%)**
≤3 session	124 (31.8%)	1–2 days per week	3 (0.8%)
4–5 sessions	113 (29%)	2–3 days per week	93 (23.8%)
5–6 session	76 (19.5%)	3–4 days per week	161 (41.3%)
≥6 sessions per week	77 (19.7%)	≥5 days per week	133 (34.1%)
**Warm-up time in each session**	**N (%)**	**Sessions in a Day**	**N (%)**
≤15 min	60 (15.4%)	1 session	145 (37.2%)
15–30 min	300 (76.6%)	2 sessions	138 (35.4%)
31–45 min	28 (7.5%)	3 sessions	96 (24.5%)
≥46 min	2 (0.5%)	≥3 sessions	11 (2.7%)
**Warm-up Method**	**N (%)**	**Duration of each session**	**N (%)**
Stretching	28 (7.2%)	≤1 h	3 (0.8%)
Running	33 (8.5%)	1–2 h	263 (67.4%)
Combat exercise	67 (17.2%)	2–3 h	110 (28.2%)
Stretching ± running	65 (16.7%)	≥3 h	14 (3.6%)
Stretching ± combat exercise	33 (8.5%)		
Stretching ± Running ± Combat Exercise	164 (42.1%)		

Abbreviation: ≥ More Than; ≤ Less Than; % Distribution Percentage; N = numbers.

## Data Availability

The data that support the findings of this study are available on request from the corresponding author, [A.L].

## Supplementary Materials

The following are available online at https://www.mdpi.com/article/10.3390/ijerph18136888/s1, File S1: Personal and sport information questionnaire, File S2: Questionnaire of Knee Injury Incidence, File S3: Knee Outcome Survey Activities of Daily Living Scale (ADLS).

## References

[1] Kazemi M., Shearer H., Choung Y.S. (2005). Pre-competition habits and injuries in Taekwondo athletes. BMC Musculoskelet. Disord..

[2] Viswanath Y.K.S., Rogers I.M. (1999). A non-contact complete knee dislocation with popliteal artery disruption, a rare martial arts injury. Postgrad. Med. J..

[3] Pieter W. (2005). Martial arts injuries. Med. Sport Sci..

[4] Caine D., Maffulli N. (2005). Epidemiology of Children’s Individual Sports Injuries: An Important Area of Medicine and Sport Science Research.

[5] Critchley G.R., Mannion S., Meredith C. (1999). Injury rates in Shotokan karate. Br. J. Sports Med..

[6] Kazemi M., Waalen J., Morgan C., White A.R. (2006). A profile of Olympic Taekwondo competitors. J. Sport. Sci. Med..

[7] Lystad R.P., Augustovičová D., Harris G., Beskin K., Arriaza R. (2020). Epidemiology of injuries in Olympic-style karate competitions: Systematic review and meta-analysis. Br. J. Sports Med..

[8] Čierna D., Lystad R.P. (2017). Epidemiology of competition injuries in youth karate athletes: A prospective cohort study. Br. J. Sports Med..

[9] Beneke R., Beyer T., Jachner C., Erasmus J., Hütler M. (2004). Energetics of karate kumite. Eur. J. Appl. Physiol..

[10] Yalfani A., Raeisi Z. (2019). Effects of Lace up Brace on the Ankle Muscles Activity in Different Foot Position during Drop Landing with and without Fatigue. New Approaches Sport Sci..

[11] Burke D.R. (1981). Treating Martial Arts Injuries.

[12] Mistry H., Connock M., Pink J., Shyangdan D., Clar C., Royle P., Court R., Biant L.C., Metcalfe A., Waugh N. (2017). Autologous chondrocyte implantation in the knee: Systematic review and economic evaluation. Health Technol. Assess..

[13] Sahebozamani M., Beyranvand R. (2016). A review of injury assessment in Iranian martial artists: Systematic review. Sci. J. Rehabil. Med..

[14] Mahdavi Mohtasham H., Shahrbanian S. (2017). An Investigation of Knee Injury Prevalence and its Mechanism among Premier League Soccer Referees in Iran. J. Sport Biomec..

[15] Naserpour H., Mirjani M. (2019). An Investigation of Ankle Injury Prevalence and Its Mechanism Among Iranian Professional Karateka. J. Sport Biomec..

[16] Arriaza R., Leyes M. (2005). Injury profile in competitive karate: Prospective analysis of three consecutive World Karate Championships. Knee Surgery Sports Traumatol. Arthrosc..

[17] Livingston L.A., Spaulding S.J. (2002). OPTOTRAK measurement of the quadriceps angle using standardized foot positions. J. Athl. Train..

[18] Dadgar H., Sahebozamani M., Noorai T., Sharifian E. (2011). The Relationship between Q—Angle and Non-Contact Injuries of Lower Extremity in Male Karate Players. Sport Med. J..

[19] Destombe C., Lejeune L., Guillodo Y., Roudaut A., Jousse S., Devauchelle V., Saraux A. (2006). Incidence and nature of karate injuries. Jt. Bone Spine.

[20] Rickham P.P. (1964). Human Experimentation Code of Ethics of the World Medical Association. Br. Med. J..

[21] von Elm E., Altman D.G., Egger M., Pocock S.J., Gøtzsche P.C., Vandenbroucke J.P. (2007). The Strengthening the Reporting of Observational Studies in Epidemiology (STROBE) statement: Guidelines for reporting observational studies. Lancet.

[22] Roos E., Roos H.P., Lohmander S., Ekdahl C., Beynnon B.D. (1998). Knee Injury and Osteoarthritis Outcome Score (KOOS)—Development of a self-administered outcome measure. J. Orthop. Sports Phys. Ther..

[23] Roos E.M., Lohmander L.S. (2003). The Knee injury and Osteoarthritis Outcome Score (KOOS): From joint injury to osteoarthritis. Health Qual. Life Outcomes.

[24] Salavati M., Mazaheri M., Negahban H., Sohani S., Ebrahimian M., Ebrahimi-Takamjani I., Kazemnejad A. (2008). Validation of a Persian-version of Knee injury and Osteoarthritis Outcome Score (KOOS) in Iranians with knee injuries. Osteoarthr. Cartil..

[25] Irrgang J.J., Snyder-Mackler L., Wainner R.S., Fu F.H., Harner C.D. (1998). Development of a patient-reported measure of function of the knee. J. Bone Jt. Surgery Ser. A.

[26] Piva S.R., Gil A.B., Moore C.G., Fitzgerald G.K. (2009). Responsiveness of the activities of daily living scale of the knee outcome survey and numeric pain rating scale in patients with patellofemoral pain. J. Rehabil. Med..

[27] Impellizzeri F.M., Mannion A.F., Leunig M., Bizzini M., Naal F.D. (2011). Comparison of the Reliability, Responsiveness, and Construct Validity of 4 Different Questionnaires for Evaluating Outcomes after Total Knee Arthroplasty. J. Arthroplast..

[28] Mazaheri M., Salavati M., Negahban H., Sohani S., Taghizadeh F., Feizi A., Karimi A., Parnianpour M. (2010). Reliability and validity of the Persian version of Foot and Ankle Ability Measure (FAAM) to measure functional limitations in patients with foot and ankle disorders. Osteoarthr. Cartil..

[29] Weigold A., Weigold I.K., Russell E.J. (2013). Examination of the equivalence of self-report survey-based paper-and-pencil and internet data collection methods. Psychol. Methods.

[30] Rossettini G., Palese A., Geri T., Fiorio M., Colloca L., Testa M. (2018). Physical therapists’ perspectives on using contextual factors in clinical practice: Findings from an Italian national survey. PLoS ONE.

[31] Piejko L., Mosler D., Grzebisz N. (2019). Sport Injuries in Karate Kyokushin Athletes. Biomed. J. Sci. Tech. Res..

[32] VencesBrito A., Rodrigues-Ferreira M., Antonio Castro M., Polak E., Valente E.J., Romero F., Figueiredo A. (2019). Sport injuries in Portuguese female and male karateka: A retrospective study. Ido Mov. Cult. J. Martial Arts Anthropol..

[33] Rahnama N., Bambaeichi E., Bagher Nazarian A., Batavani M., Sadeghipour H. (2011). Injury profile of Iranian professional male and female karate players. J. Exerc. Sci. Med..

[34] Macan J., Bundalo-Vrbanac D., Romić G. (2006). Effects of the new karate rules on the incidence and distribution of injuries. Br. J. Sports Med..

[35] Čierna D., Barrientos M., Agrasar C., Arriaza R. (2017). Epidemiology of injuries in juniors participating in top-level karate competition: A prospective cohort study. Br. J. Sports Med..

[36] Naserpour H., Mimar R., Khoshjamal Fekri S. (2017). The Effect of Eight-Weeks General Preparation Exercise on Some Selected Biomechanical, Anthropometrical and Physiological Parameters of the Iranian National Females’ Taekwondo Team. J. Sport Biomech..

[37] Sharma L., Song J., Felson D., Cahue S., Shamiyeh E., Dunlop D.D. (2001). The role of knee alignment in disease progression and functional decline in knee osteoarthritis. JAMA.

[38] Buschbacher R.M., Prahlow N.D., Dave S.J. (2009). Sports Medicine and Rehabilitation: A Sport-Specific Approach.

[39] Chen J., Kim J., Shao W., Schlecht S.H., Baek S.Y., Jones A.K., Ahn T., Ashton-Miller J.A., Holl M.M.B., Wojtys E.M. (2019). An Anterior Cruciate Ligament Failure Mechanism. Am. J. Sports Med..

[40] Petushek E.J., Sugimoto D., Stoolmiller M., Smith G., Myer G.D. (2019). Evidence-Based Best-Practice Guidelines for Preventing Anterior Cruciate Ligament Injuries in Young Female Athletes: A Systematic Review and Meta-analysis. Am. J. Sports Med..

[41] Skinner H.B., Barrack R.L. (1991). Joint position sense in the normal and pathologic knee joint. J. Electromyogr. Kinesiol..

[42] LaBella C.R., Huxford M.R., Grissom J., Kim K.Y., Peng J., Christoffel K.K. (2011). Effect of neuromuscular warm-up on injuries in female soccer and basketball athletes in urban public high schools: Cluster randomized controlled trial. Arch. Pediatr. Adolesc. Med..

[43] Wu H., Hackett T., Richmond J.C. (2002). Effects of meniscal and articular surface status on knee stability, function, and symptoms after anterior cruciate ligament reconstruction: A long-term prospective study. Am. J. Sports Med..

