# Supplementary material for: An Investigation of Knee Injury Profiles among Iranian Elite Karatekas: Observations from a Cross-Sectional Study
Source: Int J Environ Res Public Health. 2021 Jun 27;18(13):6888. doi: 10.3390/ijerph18136888 (PMC8296942; doi:10.3390/ijerph18136888)
Supplement: Supplementary file 1 [file ijerph-18-06888-s001.zip › A-NO=1.pdf]

Questionnaire code=

Personal and sport information questionnaire

Name = age =

Weight = height =

Please mark the best answer

*How many day do you work out in week?
A. 1-2 days
B. 2-3 days
C. 3-4 days
D. more than 5 days

*How many day do you work out in week?
A. up to 3 sessions
B. 4-5 sessions
C. 5-6 sessions
D. more than 6 session

*How long does your training session last?
A. less than 1 hour
B. 1-2 hours
C. 2-3 hours
D. more than 3 hours
*At which level do you practice?
A. National
B. Clubs
C. Colligate

* How many years have you been seriously exercising?
A. Less than 3 years
B. 3-5 years
C. More than 5 years

* do you do warm up exercises before combat practicing?
A. Yes
B. No
If you answered yes please answer following question

*how long does it takes to warm –up?
A. Less than 15 minute
B. 15-30 minute
C. 31-45 minute
D. More than 46 minute

Questionnaire code=

* Which type of warm-up exercises do you use?
A. Stretching
B. Running
C. Combat exercise
D. Stretching ± running
E. Stretching ± combat exercise
F. Stretching ± Running ± Combat Exercise
