# Supplementary material for: An Investigation of Knee Injury Profiles among Iranian Elite Karatekas: Observations from a Cross-Sectional Study
Source: Int J Environ Res Public Health. 2021 Jun 27;18(13):6888. doi: 10.3390/ijerph18136888 (PMC8296942; doi:10.3390/ijerph18136888)
Supplement: Supplementary file 1 [file ijerph-18-06888-s001.zip › B- NO=2.pdf]

Questionnaire code=

Questionnaire of Knee Injury Incidence

Please mark the best answer

*. Do you have History of Knee injury?

A. Yes

B. No

** If you answer yes please answer the following question

*Which knee was effected?

A. Dominant one

B. None dominant one

C. Both

* When did your knee injury occur?

A. Training

B. Match

* did you see dr or physiotherapy after that?

A. Yes

B. No

* If you see a doctor or physiotherapist, which part of your knee was injured by his/her diagnosis?

A. ACL	B. PCL
C. MCL	D. LCL
E. Meniscus	F. Knee Dislocation
J. Patella Dislocation	K. patellar tendinitis
L. cartilage damage	M. Patellofemoral Pain
N. NO Refer	O. Without any

*. Which mechanism cause the injury?

Questionnaire code=

A. Landing from jump
B. abrupt stop
C. opponent hitting
D. hitting the opponent
E. twist on stable leg
F. falling
G. others

* Which type of treatments do you use?

A. No treatment
B. RICE (Rest, Ice treatment, Compression, Elevation)
C. Physiotherapy
D. medical treatments like pills
E. brace or cast
F. surgery
G. other

* how long does it take to back to sport?

A. 1-7 days
B. 8-20 days
C. More than 21 days
