# Supplementary material for: An Investigation of Knee Injury Profiles among Iranian Elite Karatekas: Observations from a Cross-Sectional Study
Source: Int J Environ Res Public Health. 2021 Jun 27;18(13):6888. doi: 10.3390/ijerph18136888 (PMC8296942; doi:10.3390/ijerph18136888)
Supplement: Supplementary file 1 [file ijerph-18-06888-s001.zip › C-NO-3.pdf]

Knee Outcome Survey Activities of Daily Living Scale (ADLS).

Symptoms: To what degree does each of the following symptoms affect your level of activity? (check one answer on each line)

	I do not have the symptom	I have the symptom, but it does not affect my activity	The symptom affects my activity slightly	The symptom affects my activity moderately	The symptom affects my activity severely	The symptom prevents me from all daily activity
Pain						
Stiffness						
Swelling						
Giving way, buckling, or shifting of the knee						
Weakness						
Limping						

Functional Limitations With Activities of Daily Living: How does your knee affect your ability to: (check one answer on each line)

	Activity is not difficult	Activity is minimally difficult	Activity is somewhat difficult	Activity is fairly difficult	Activity is very difficult	I am unable to
Walk						
Go up stairs						
Go down stairs						
Stand						
Kneel on front of your knee						
Squat						
Sit with your knee bent						
Rise from a chair						

Scoring: The first column is scored 5 points for each item, followed in successive columns by scores of 4, 3, 2, 1, and 0 for the last column. The total points from all items are summed, then divided by 70 and multiplied by 100 for the ADLS score. For example, if the individual places marks for 12 items in the first column, and 2 items in the second column the total points would be 12x5 = 60 points, plus 2 x 4 = 8 points, for a total of 68 points. The ADLS score would then be 68/70 x 100 = 97%.

Global Rating: How would you rate your level of functioning during your usual daily activities on a scale from 0 to 100 with 100 being your level of function prior to your knee problem and 0 being the inability to perform any of your usually daily activities? ___ / 100 %

<u>Knee Outcome Survey Sports Activities Scale (SAS)</u>

<u>Symptoms:</u> To what degree does each of the following symptoms affect your level of sports activity? (Check ONE answer to each line)

	Never have	Have, But does not affect my sports activity	Affects my sport activity slightly	Affects sports activity moderately	Affects sports severely	Prevents me from all sports activity
**Pain**						
**Grinding or grating**						
**Stiffness**						
**Swelling**						
**Slipping or partial giving way of knee**						
**Buckling or full giving way**						
**Weakness**						

Functional Limitations with Sports Activities: How does your knee affect your ability to: (Check ONE answer on each line)

	Not difficult at all	Minimally difficult	Somewhat difficult	Fairly difficult	Very difficult	Unable to do
**Run straight ahead**						
**Jump and land on your involved leg**						
**Stop and start quickly**						
**Cut and pivot on your involved leg**						

<u>Scoring:</u> The first column is scored 5 points for each item, followed in successive columns by scores of 4,3, 2, 1, and 0 for the last column. The total points from all items are summed, then divided by 55 and multiplied by 100 for the SAS score. For example, if the individual places marks for 9 items in the first column, and 2 items in the second column the total points would be 9x5 = 45 points, plus 2 x 4 = 8 points, for a total of 53 points. The SAS score would then be 53/55 x 100 = 96%.
